# Distributed Thermal Monitoring of High-Voltage Power Lines

**DOI:** 10.3390/s23052400

**Published:** 2023-02-21

**Authors:** Levente Rácz, Dávid Szabó, Gábor Göcsei, Bálint Németh

**Affiliations:** Department of Electric Power Engineering, Faculty of Electrical Engineering and Informatics, Budapest University of Technology and Economics, Műegyetem rkp. 3., H-1111 Budapest, Hungary

**Keywords:** power line monitoring, sensors, thermal sensors, high-voltage lines, distribution sensor placement strategy, hardware development

## Abstract

The purpose of this paper is to present the sensor placement strategies that currently determine the thermal monitoring of the phase conductors of high-voltage power lines. In addition to reviewing the international literature, a new sensor placement concept is presented based on a strategy centered on the following question: What are the chances of thermal overload if devices are only placed in certain tension sections? In this new concept, the number and installation location of the sensors are determined in three steps, and a new type of tension-section-ranking constant is introduced that is universal in space and time. The simulations based on this new concept show that the data-sampling frequency and the type of thermal constraint influence the number of sensors. The paper’s main finding is that there are cases when only a distributed sensor placement strategy can result in safe and reliable operation. However, due to requiring a large number of sensors, this solution means additional expenses. In the last section, the paper presents different possibilities to reduce costs and introduces the concept of low-cost sensor applications. These devices can result in more flexible network operation and more reliable systems in the future.

## 1. Introduction

**Figure 1:**
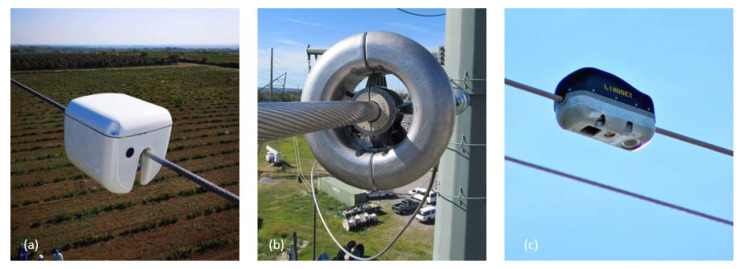
Direct conductor temperature measurement sensors: (**a**) Gridpulse-Base sensor [11]; (**b**) Power Donut sensor [12]; (**c**) Lindsey-TLM sensor [13].

The transformation of the power system also means an appreciation of the role of the transmission network [1]. The operational strategies of transmission system operators (TSOs) are currently more complex than a few decades ago—in many cases, they have to deliver increased power on aging networks [2]. However, while previously, it was sufficient to monitor the power flowing through the phase conductor and the thermal response at the substation, in the future, a more extensive set of actual measurements will be required for full-scale commissioning [3]. Recently, monitoring the thermal behavior of transmission lines, which requires an extensive equipment background, has become an increasingly central issue [4]. The range of applications of line-monitoring sensors is extensive; each device can be used for several purposes on high-voltage networks [5]. We can distinguish three main groups of strategies for sensor application: extended line rating, asset management, and reduced risk caused by extreme weather phenomena. The essence of dynamic line rating (DLR) is to use sensors and weather stations to adjust the ampacity of phase conductors to changes in the environment, which results in an average of 20-30% extra ampacity on a transmission line 95% of the time [6,7]. This excess transfer capacity can be used well during congestion management and when increasing the flow of power on cross-border lines, which results in more balanced energy prices and safer transmission [3,8]. Asset management is essential because the expansion of the transmission network is costly, and the aging of network elements can pose severe problems in terms of reliable power supply and safe and secure operation [8]. Protection against extreme weather is essential due to global warming and the resulting extreme events that arise (power line icing, forest fires, etc.) [9,10]. The common feature among all three strategies is that they are closely related to the thermal conditions of phase conductors, which requires a well-structured monitoring strategy in addition to the appropriate tools. This paper focuses on DLR sensor applications and thermal monitoring purposes. Figure 1. presents examples of these devices, some of themcan be applied for the other two purposes, too.

The essence of the DLR method is that it always determines a transfer capacity limit on phase conductors that, together with the thermal effects from the environment, results in the maximum conductor temperature. Assuming that DLR is used on a transmission line to fully utilize the transfer capacity, ideally, the conductor always operates around its thermal limit. This means that in such cases, it is advisable to focus on temperatures above 40 °C and not to examine the range below room temperature.

## 2. Sensor Placement Strategies

While proper line monitoring is paramount, there are still open questions waiting for the consensus of researchers and sensor manufacturers [5]. First, different approaches are used in a broad range of sensors available in the market that measure various parameters as inputs for line rating [14]. Sometimes, the end users need to define which solution fits their system and operational conditions the best. Even if the appropriate sensor type is chosen, their installation may raise essential questions, such as the necessary sensor number and the unit’s location on the power line [5,15]. Bad decision-making on these factors can significantly impair the line-monitoring system’s economic benefit and technical accuracy [16].

In the USA, at the end of the 1980s, researchers started investigating which parameters affect the number of line-monitoring sensors [17]. At that time, weather parameters, conductor properties, and load current were identified among the data called sensitivity parameters. Later, CIGRE brochures mentioned that the line length, the number of surroundings, and the climatic environment homogeneity are also among the criteria [18]. The weather parameters’ spatial distributions are essential, as this can cause various thermal behaviors in power line conductors [5,19,20]. It is vital to mention that the generated heat is hardly transferred in the longitudinal direction, which can result in a longitudinal temperature variation [21]. In natural conditions, this phenomenon was investigated for several transmission lines and simulated via physical and thermal models with different results [18].

Due to the lack of complex installation strategies, the initial monitoring projects often followed the rules of thumb when installing line-monitoring sensors [22]. Some of these were based on the experience of historical distributions of weather parameters. Thus, sensors were placed in sections with low or high wind speeds [23]. Others followed different strategies, such as equidistant sensor placement, which refers to several sensors placed at equal distances [22]. Later, these approaches were mostly found to need more accuracy. Soon after, research was initiated in which transmission lines’ thermal weak points were defined as “critical spans” [17]. Based on this theory, it is not the entire transmission line but only the critical parts that need to be monitored, leading to significant cost savings [18]. The main question in the case of critical spans is what the term exactly means in different studies and how to find them [16].

In the late 1980s, researchers defined those spans as critical in which the temperature was the highest [17]. They found that the number of critical spans is mainly influenced by weather parameters, with the dominant influence of the wind. An initial approximation in calm weather was to place sensors at 1–2 miles (1.5–3 km) along the transmission line. Under higher wind loads, the measuring units can be placed further apart. However, in this model, it should be considered that the span with the highest temperature is not always critical from an operational point of view. For many transmission lines, the thermal limit is not caused by annealing but by sag-clearance relations. Thus, it is not the hot spots but the spans where the minor clearance reserve occurs that need to be identified [18].

In the 1990s, studies showed that locally measured conductor temperatures have a limited relationship with sagging [24,25]. Because the tensions are equalized within a given tension section, it is not necessary to focus on the local temperatures; rather, the focus should be on the average temperature. This principle was also followed by CIGRE, which emphasized that the average temperature variation between line sections is also essential. However, these approaches only focused on sagging and ignored that each tension section and span is unique [18].

In 2010, a new study dealt with the critical span issue in more detail [22]. It suggested defining buffer zones from the current and permissible sags and converting them to a current dimension. Based on this concept, line rating becomes a minor feature of the calculated currents. Regarding dead-end sections, it was recommended to use the ruling span method (the assumption of equally long spans in a dead-end section) in which tensions and sags are equal. This model suggested that it is not practical to equip every span with a monitoring device. A sufficient number of sensors need to be installed to avoid clearance problems.

While most critical span analyses focused on the sag issue in the spans, other papers followed a different approach [26]. The main goal was to detect critical parts from the annealing and aging points of view, which are strictly linked to thermal hot spots.

In 2012, a sensor placement strategy emerged that was significantly different from previous approaches [27]. In that concept, a heuristic model based on historical WFR weather data was used to determine the number of sensors and their installation location with the Pearson correlation rate. Compared with the equidistant strategy, this method reduced the required sensors. The model’s weakness was that if the desired correlation level was reached too early, the simulation could skip spans even if they were critical.

Later, another study further developed this model by including sag conditions [28]. This model defined critical spans as sections with a lower thermal capacity due to unfavorable weather conditions. This approach already included annealing and represented several critical spans in a tension section. However, this model worked with interpolated weather data and did not consider the cooling effect of precipitation. In later research, it was demonstrated that the interpolation of weather parameters could cause a significant error in line rating and the conductor’s temperature calculations [15]. Weather parameters were eliminated with static analysis in time [29]. It was also highlighted that not only ground clearance but also the objects under the line need to be investigated.

It is also worth mentioning that a novel direction in the critical span analysis includes critical periods in the simulations [30]. 

During the evolution of sensor placement strategies, it can be observed that although many models made a valuable contribution to more accurate line monitoring, they were not able to integrate all the existing parameters into one system. The models precisely based on hot-spot analysis did not consider sag-clearance issues. At the same time, precise clearance-reserve-based systems that handle weather variations along the transmission line ignored the annealing caused by high local temperatures. In addition, none of the models considered that each transmission line has unique tension section parameters and that there may be other limiting factors (electric field, aging of the conductor, etc.) that can also significantly change the number of sensors. A model that combines adequate features from operational aspects can lead to reliable DLR systems [16].

## 3. Novel Distributed Sensor Placement Protocol

The motivation for the novel sensor placement protocol is based on the insufficiency of the existing models [16]. The main goal during the development of this new protocol was to combine the advantages of the previous models and, simultaneously, provide the end user with helpful information from a practical point of view even before the installation [16].

The core feature of the proposed protocol is to avoid the real risk of thermal overload. The new protocol was built on the following question: If we monitor only dedicated sections on the line, how likely is the thermal overload occurrence in other unmonitored areas [16]? The protocol’s purpose is to supply the transmission line with as many line-monitoring devices as possible to guarantee safe operation in terms of thermal conditions at a given risk level. This is a fundamental difference from previously used approaches. 

The proposed sensor installation protocol can be separated into the following three main steps [16]:

- Step (1)—general power line analysis, including the first sensor’s position (line-monitoring sensor and weather station);
- Step (2)—definition of the thermal limiting factor: clearance, annealing, or both;
- Step (3)—risk management due to weather parameter changes along the line considering the unique attributes of the tension sections.

### 3.1. Step 1 of the Distributed Sensor Placement Concept

**Figure 2:**
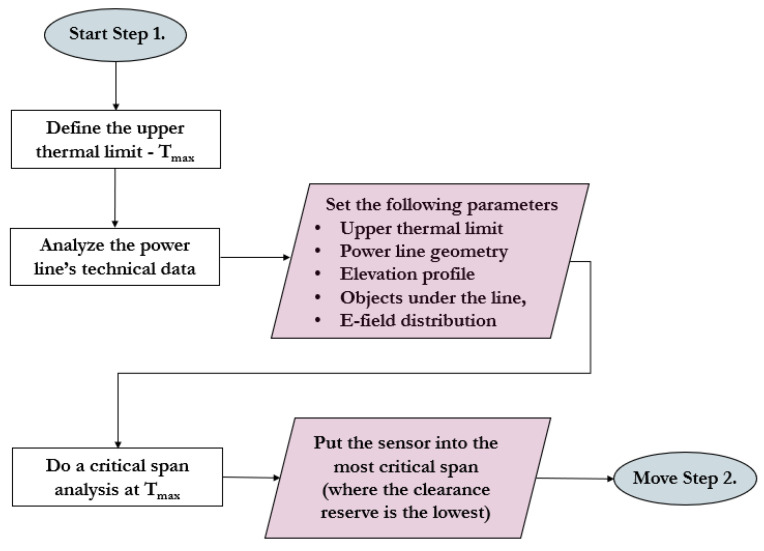
Flowchart of Step 1 in the novel sensor placement protocol based on [16].

In Step 1 (detailed in Figure 2), the whole sensor placement protocol should start with the analysis of the technical properties of the line, such as the applied voltage level, safety distances, ampacity, etc [16]. 

After that, a so-called critical span analysis must be performed on the whole power line without any weather parameters. The transmission line must be split into tension sections, and the catenary curve must be simulated at the maximum conductor temperature according to Equations (1)–(5) [16,31].

(1) $$y=p\;\cdot \frac{e^{\frac{x}{p}}+e^{-\frac{x}{p}}}{2}=p\cdot \;ch\frac{x\;\gamma }{\sigma_{h}}\;$$

where *y* represents the shape of the catenary curve, *p* is the curve’s parameter, *γ* is the weight force for a cross-section of 1 mm^2^ of a 1 m long conductor, and *σ_h_* is the horizontal component of the tensile stress. 

The catenary curve’s parameter is defined using Equation (2).

(2) $$p=\frac{\sigma_{h}\;}{\gamma }$$

The sagging of the catenary curve can be calculated with Equation (3).

(3) $$b_{h}=\frac{\sigma_{h}}{\gamma }\;\left[ch\frac{a\;\gamma }{2\;\sigma_{h}}-1\right]$$

Given Equation (4), Equation (3) can be modified to Equation (5), which is the standard formula to determine the sag.

(4) $$ch\;2z-1=2\;sh^{2}z$$

(5) $$b_{h}=\frac{2\sigma_{h}}{\gamma }sh^{2}\frac{a\gamma }{4\sigma_{h}}$$

Knowing the elevation profile, the clearance becomes available at each tension section. The background of this resolution is twofold. One aspect is that several technical parameters are tension-span-specific in the simulation. Another factor is that tension sections represent separate units regarding the wind direction [16,18]. 

During the analysis, attention should be paid to the electric field distribution and the objects under the transmission line, which may cause a lower clearance value than the ground clearance itself [16]. The tension sections’ span with the lowest clearance reserve should be chosen for the first line-monitoring sensor. At this point, the extent of the transmission line is already considered in a technical sense [16].

### 3.2. Step 2 of the Distributed Sensor Placement Concept

**Figure 3:**
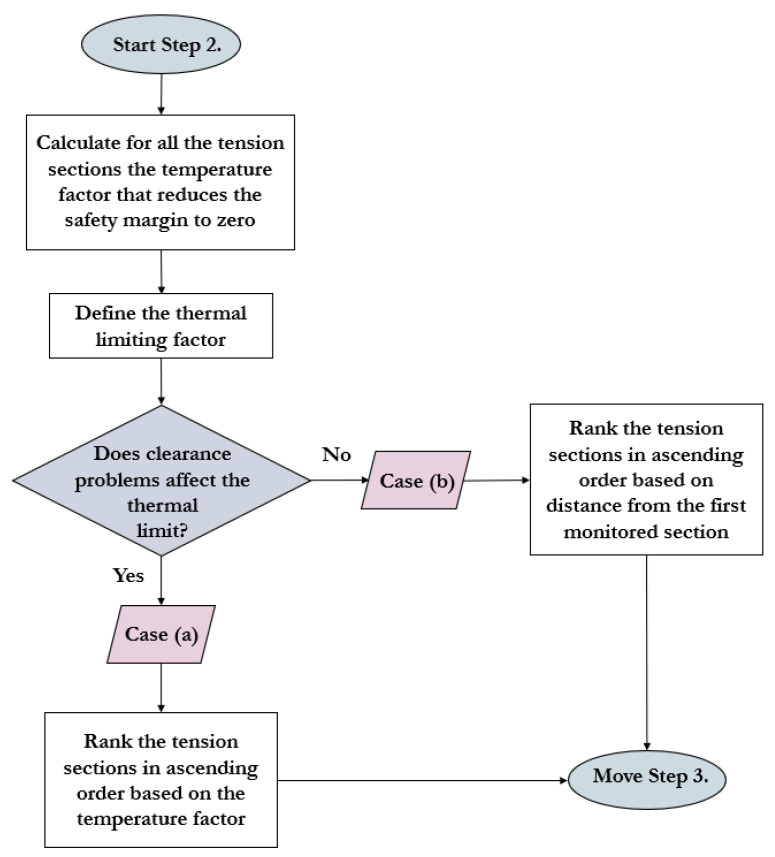
Flowchart of Step 2 in the novel sensor placement protocol based on [16].

Step 2 (presented in Figure 3) involves the determination of the upper thermal limit of the power line and the factors that play a role in this determination. 

Two prominent different cases can be defined because of thermal overload [16]:

- Case (a): sag-clearance problem (mostly 40–60 °C is the upper thermal limit of the power lines);
- Case (b): annealing of the conductor (mostly 80 °C or higher temperature is the upper limit of the power lines).

The power lines in which both clearance and annealing pose a risk should be handled as power lines of Case (a). It is essential to note that conductor temperature monitoring with sensors is vital for a comprehensive and secure DLR system in both cases [16].

This is necessary because, in a tiny percentage of cases, one line-monitoring sensor is sufficient to inspect the entire transmission line. For this reason, tension sections must be prioritized to select the location of the following sensor or sensors. This ranking is carried out differently for Case (a) and Case (b) [16].

In Case (a), the main criterion comes from maintaining the clearance, so the most significant emphasis should be placed on this factor when ranking the power line sections. In early models, the location of the sensors was chosen based on the span where the hot spots were most likely to occur. However, these solutions did not focus on the proper parameter. In some other studies, the sections were ranked based on the line rating calculated from environmental parameters [27]. Still, at the same time, they did not consider that each span, and therefore, each tension section, is also unique. This also means that some sections have more clearance reserve than others when installed [22]. Those approaches that focus on the clearance reserve value do not consider that this parameter is time-dependent and can change depending on environmental parameters [32]. This is supported by previous experiences that indicate that critical sections shift in time and space [16,30]. 

For this issue, the goal was to introduce a new section-ranking constant over time that can be used universally regardless of environmental changes. Therefore, in Case (a), the result of the critical span analysis must be used as a starting point. Based on the necessary clearance limit and the results of the critical span analysis, the value of the clearance reserve at the maximum temperature is clearly defined in each tension section. This distance shows how much margin is left in the span at the maximum conductor temperature. If this reserve is higher than zero under the investigated section, the clearance problem may not occur despite the high temperature [16]. 

This novel concept converts these clearance reserve values (distance-type quantity) into a so-called temperature factor (temperature-type quantity) [16]. The temperature factor of each section is the temperature value at which the clearance reserve becomes zero. Applying these temperature factors to the ranking method of the tension sections has the following features [16]:

- It is universal (not time-dependent);
- It considers the unique attributes of each span;
- It considers the clearance relations.

In Case (a), if it is necessary to install additional sensors (detailed in Step 3), the next ranked section must be equipped with a line-monitoring device [16].

In Case (b), annealing is the critical factor. Thus, a different strategy must be followed. There is no need to rank the tension spans since exceeding the maximum temperature instantly raises the risk at all the tension sections. Suppose that hot-spot analysis can be performed for any section of the power line. In that case, additional sensors should be placed onto the hot-spot sections. Otherwise, an iterative approach is recommended. ‘N’ is the number of sensors that divide the power line into ’N+1’ sections. The following sensor should permanently be installed at the midpoint of the most extended unmonitored transmission line section until reaching the required criteria (detailed in Step 3) [16].

### 3.3. Step 3 of the Distributed Sensor Placement Concept

**Figure 4:**
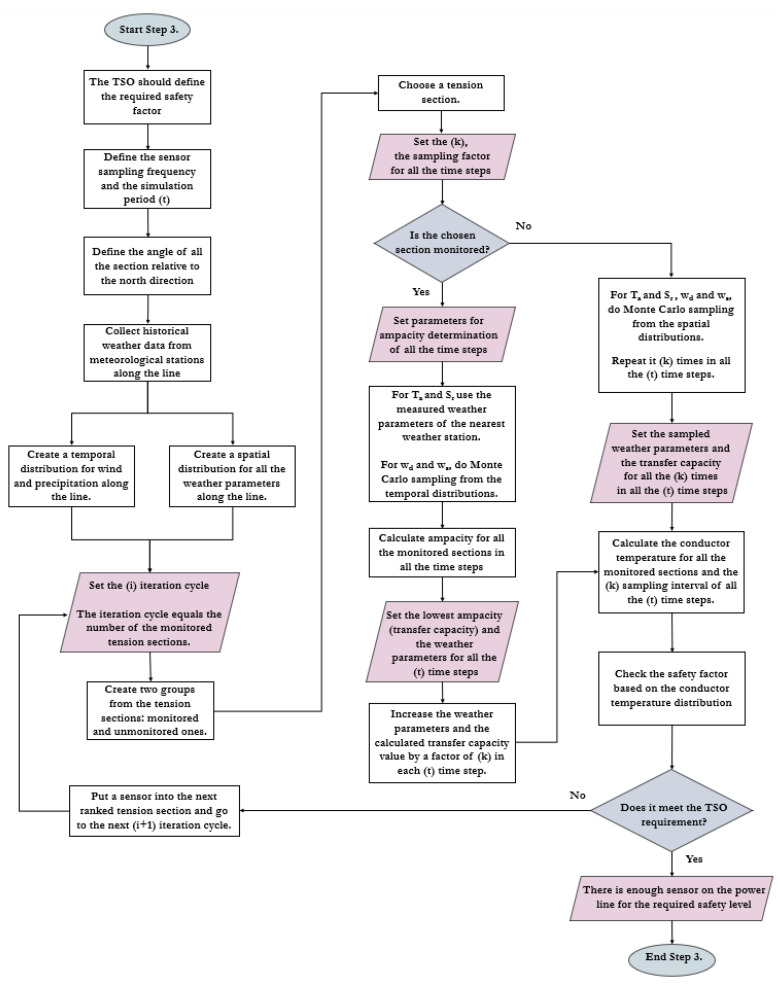
Flowchart of Step 3 in the novel sensor placement protocol based on [16].

Step 3 (detailed in Figure 4) reveals the environmental parameters’ variability in time and space around the power line. This is relevant since they can cause severe risk in the unmonitored tension sections [16].

Step 3 begins with the TSO defining the risk factor still permissible for them in regular operation. After that, it is necessary to determine the interval at which the sensor sends data and the sampling frequency. This is usually 10–15 min, but a longer interval can also occur. Considering that the wind direction also plays a significant role in the thermal model used by CIGRE, for each tension section of the transmission line, the azimuth angle close to the north direction must be defined [21]. This is necessary because the direction of the wind also has an effect on the conductor temperature. When generating or measuring a wind direction value, it is always necessary to define the angle relative to the main direction, which, in practice, is usually the north one. Defining the azimuth angle of the transmission line’s tension section from the north direction is important because this can help to reveal the real cooling effect of the wind of each section. Ignoring it causes errors in the thermal model.

Historical weather patterns along the transmission line must then be collected. There are two aspects to this since the spatial distribution of each time-varying parameter must be mapped, as well as its temporal distribution. Knowing the historical weather patterns, it is possible to determine which distribution and parameters can be fitted to a given parameter (wind speed, wind direction, precipitation, etc.). Generally, the Weibull distribution can be applied to the wind speed, while the von Mises distribution can be used for the wind direction. In this way, the spatial and temporal distribution of individual parameters can be created, from which samples can already be taken for each examined time step. From here, an iteration cycle can be used to determine in which tension sections thermal overload may occur depending on the number of sensors used [16].

In the first iteration step, we assume sensors are only in the tension section defined in Step 1. In this case, the data of the nearest national weather station are used for this section during the set’s test period. In the case of wind (speed and direction) and precipitation, Monte Carlo sampling is performed from a distribution based on historical patterns, assuming that they are random variable parameters. With the help of the weather data, a transfer capacity value can be calculated for each moment in time. The ampacity calculation is detailed in Equation (6) [21].

(6) $$I=\sqrt{\frac{P_{c}\left(T\right)+P_{r}\left(T\right)-P_{s}-P_{M}\left(T,I\right)}{R_{AC}\left(T\right)}}\;$$

where *I* is the ampacity (A), *P_S_* is the solar heating (W/m), *P_M_* is the magnetic heating (W/m), *P_C_* is the convective cooling (W/m), *P_r_* is the radiative cooling (W/m), and *R_AC_* is the AC resistance of the conductor (Ω).

Since sensor measurement is only performed in this section, its ampacity must be valid for the entire transmission line. Then, it is necessary to examine when the application of this ampacity causes a conductor temperature increase in other tension sections. During the thermal simulation of the subsequent tension sections, the ambient temperature, solar radiation, and current are assumed to be nearly constant. Wind (speed and direction) and precipitation data are sampled using Monte Carlo simulation from the spatial distribution functions. The result is a conductor temperature distribution that shows the thermal conditions of the transmission line as a whole during the examined period. Then, it is necessary to check how much the simulated conductor temperature values will be greater than the temperature factor of the next ranked tension section. When determining the risk, Case (a) and Case (b) differ. In the case of Case (a), only the temperatures higher than the temperature factor of the given section pose a risk. By contrast, in Case (b), any temperature above 100 °C means potential danger. The risk factor (RF) can thus be determined with Equation (7).

(7) $$RF=P\left(T_{c\;simulated}>T_{max}\right)=\frac{\sum_{i=1}^{n}N_{fi}}{N}$$

where *T_c_* is the simulated conductor temperature (°C), *N_fi_* is the number of simulated conductor temperatures higher than the temperature factor (-), *N* is the number of simulations (-), and *n* is the number of tension sections (-).

The risk factor must be compared with the limit value defined by the TSO, where an unfavorable result means the necessity of the sensor monitoring of the next ranked section. This is how the cycle starts again but with two monitored sections. In that case, the smaller ampacities calculated for the two sections must always be chosen, which will reduce the thermal risk at the end of the iteration cycle [16].

(8) $$I_{line}=\min\left(I_{t,1},\;I_{t,\;2}\right)$$

where *I_t,1_* is the ampacity of the first-ranked tension section in the *t* time interval (A), and *I_t,2_* is the ampacity of the second-ranked tension section in the t time interval (A).

This iteration must be continued until the risk factor drops below the desired value. For transmission lines in which both clearance and annealing are potential risks, the presented iteration must be performed for both Case (a) and Case (b). In this case, the one that is more favorable from a security point of view and recommends more sensors shall be chosen [16].

## 4. Operation of the Novel Concept and Discussion of the Results

**Figure 5:**
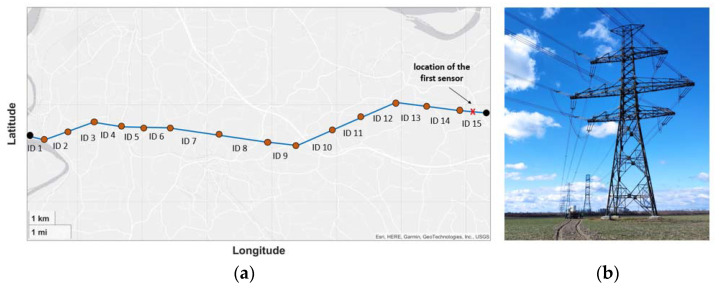
The case study of a 220 kV power line: (**a**) tension sections of the line with IDs; (**b**) image of the tension section with ID 15.

To demonstrate the advantages of this novel distributed sensor placement protocol, its operation is presented through a case study. A 220 kV double circuit power line (Figure 5) was chosen for the simulations with a T_max_ of 80 °C. This also meant that both clearance and annealing issues had to be considered for the implementation of line monitoring.

Annealing is usually the most dangerous at the locations with the highest conductor temperature, so great emphasis must be placed on local temperature measurement. As a result, it is necessary to use a line-monitoring sensor suitable for local contact temperature measurement. Devices of this type are sensors that can be installed on the phase conductor. In this application, a particular monitoring device was selected to measure local temperature and inclination, so sag-clearance conditions were also monitored. 

It is essential to note that the selected power line was already equipped with one line-monitoring sensor for demonstration purposes. The aim of the simulations was, on the one hand, to examine whether one sensor was sufficient for the transmission line, and on the other hand, to examine where and how many sensors the new protocol would recommend installing for full security.

### Simulation Results Based on the Novel Concept

In Step (1), a critical span analysis was performed for all the tension sections at 80 °C.

**Figure 6:**
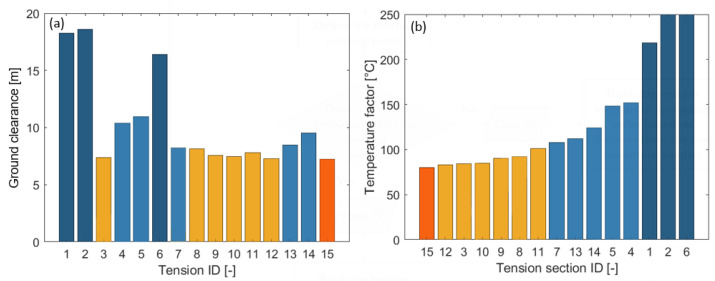
Power line section details: (**a**) ground clearance result of the critical span analysis; (**b**) ranking of the tension section based on the temperature factor [16].

During the simulations, it was found that this electric field did not cause problems, so there was no need to reduce the T_max_. The result of the critical span analysis with the ground clearances can be seen in Figure 6a.

As shown in Figure 6a, the section with ID 15 had the lowest clearance, which hardly exceed the 8 meters required by the standard. Therefore, that section had to be equipped with the first sensor. 

The next step was the identification of the thermal limiting factor. Given that the T_max_ value of the transmission line was 80 °C, an annealing problem could potentially appear. However, the line affects populated areas, forcing the clearances to be controlled. Therefore, the simulation was continued according to Case (a). Figure 6b represents the ranking of the tension sections based on the temperature factor. 

Based on Figure 6b, the first sensor must be installed in the last tension section (ID 15). If additional devices are needed, their installation order must follow the ranking of Figure 6b.

During the simulations, the following assumptions were applied:

- The safety factor (100—risk factor (%)) defined by the TSO was 99.5% for both clearance and annealing;
- To decrease the probability of conductors’ annealing, the limit temperature was set to 95 °C;
- In the case of sensor sampling, three cases were defined: data recording frequencies of 10, 15, and 20 min. A data-sampling frequency of 10 and 15 min can be expected for the sensors available on the market, so using them can make the result independent of the sensor type. The 20 min sampling is advantageous because it can be used to model if the sensor cannot send data with the preset frequency;
- The period examined in the simulation contained 4420 weather events;
- Due to the shortness (approx. 24.5 km) and orientation of the transmission line, the tension sections’ direction was omitted. The ambient temperature and solar radiation were constant in space;
- When calculating the ampacity and conductor temperatures, the cooling effect of precipitation and the current carrying capacity of substation devices was not considered;
- The only space-variable parameter was wind speed. Over one year, it was found that the wind speed distribution along the transmission line follows a Weibull distribution;
- The parameters of the probability density functions were 3.36 (κ) and 1.64 (λ). In the case of the spatial distribution of the wind speed, the same Weibull distribution was applied as a worst-case scenario;
- It was assumed that the weather and load values sampled by the sensor remain constant in the examined sampling time range.

**Figure 7:**
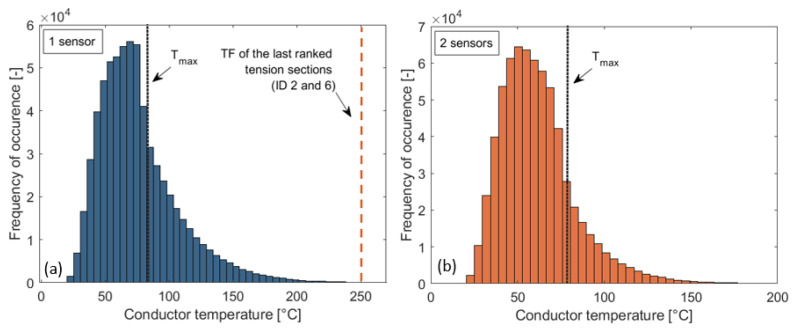
Conductor temperature distribution along the transmission line during the examined period with 10 min sensor-sampling frequency: (**a**) applying one sensor; (**b**) applying two sensors [16].

Based on Step 3 of this novel protocol, the application of only one sensor was investigated in the first iteration cycle. Figure 7a shows how the conductor temperature distribution along the transmission line developed during the analyzed period. 

It can be seen from Figure 7a that the conductor temperature several times exceeded 80 °C (T_max_), 95 °C (the annealing limit temperature), and 83 °C, which was the second-ranked tension-section temperature factor. Thus, using one sensor was not enough; additional devices were needed. Figure 7b shows how the conductor temperature distribution developed in the second iteration cycle when two sensors were used. By analogy, the simulations were continued until the desired safety factor.

**Figure 8:**
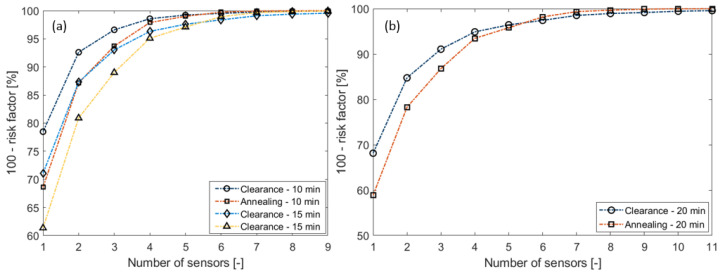
The safety factors for clearance and annealing as a function of the sensor number: (**a**) for the sampling frequency of 10 and 15 minutes; (**b**) for the sampling frequency of 20 min [16].

Figure 8 shows the safety factor values for clearance and annealing for the three sampling frequencies. While Figure 8a details the 10 min and 15 min sensor-sampling-rate cases, Figure 8b details the 20 min sensor-sampling results. 

Table 1 presents the safety factor changes as a function of the number of sensors and sampling frequency.

These simulations show that 6 devices were required for 10 min sampling, 9 devices for 15 min sampling, and 11 devices for 20 min sampling to ensure a 99.5% safety factor compared with the currently used one line-monitoring sensor. For each scenario, a green background in Table 1 indicates those sensor numbers that provide an acceptable safety level.

## 5. Discussion

Based on the results, several conclusions can be made that may influence the placement strategy of future line-monitoring sensors.

The first observation relates to monitoring the tension sections with a high-temperature factor. It can be seen from Figure 7a that the temperature does not exceed 250 °C even in the most unfavorable case, so it is not economical to install a sensor in those sections that have a higher temperature factor than 250 °C. On this transmission line, these were the sections with ID 2 and ID 6.

The second observation concerns the effect of the type of thermal constraint on the sensors. The simulations show that for this power line, avoiding annealing at all three sampling frequencies results in more sensors than the avoidance of clearance problems.

The third observation is related to the basis of sensor installation strategies. Based on the presented simulation, even in the case of short transmission lines, a distributed sensor installation concept may be needed to ensure the safe operation of the grid. This goes against the current practice, which suggests that the sensor monitoring of a few critical points is sufficient for the complete monitoring of high-voltage power lines.

Based on these three observations, in the future, TSOs must precisely define the technical specification of the sensor to be used and consider distributed sensor monitoring to ensure safe operation. A good solution for this could be applying the presented sensor placement concept. At the same time, it is essential to point out that the technical and environmental data that would allow the presented analysis are unavailable in many cases. In such cases, installing line-monitoring sensors in each tension section may be advisable. Whichever solution the TSO chooses, it must expect increased costs due to the increased number of devices.

### 5.1. Possibilities to Reduce the Monitoring Costs

There are several options for reducing the costs caused by the increased number of sensors. One solution is to reduce the number of devices placed on the power line in such a way as to maintain the conditions for safe operation. A good solution for this could be the development of DLR models that do not require the installation of weather stations [16]. The number of line-monitoring sensors can be reduced by training an artificial neural network based on the measurements of the installed sections [16]. After its calculated accuracy reaches the accuracy of the sensor measurement, the device is moved to other tension sections. With both solutions, the costs of line monitoring can be significantly reduced.

The other possible way is to design sensors that can be produced at a low cost. In this way, the number of sensors does not change on the transmission lines; only the capital is required to construct the line-monitoring ranger. The rest of the paper presents a solution from the device’s design to the test phase.

### 5.2. Concept of a Cost-Effective Line-Monitoring Sensor

For sensor placement strategies requiring many sensors for safe and reliable operation, a possible solution is using cost-effective line-monitoring equipment in the implementation phase. The motivation during cost-effective DLR sensor development is to achieve a device with only one functionality—the conductor temperature measurement—and in this way, reduce both the development cost of the sensor and the purchase price of that during a DLR system’s implementation. 

Four different workflows must be distinguished when developing such a sensor unit: sensor housing, temperature sensing, energy harvesting system, and developing a data center control unit. 

In designing sensor housing, the primary consideration is to develop a lightweight sensor that withstands extreme environmental conditions. At the same time, serial production is beneficial for its implementation in both small and large series. By summarizing these requirements, the 3D printing technique seems a proper direction due to available experience in 3D design and the wide range of raw materials.

In terms of temperature measurement, low-cost devices can also provide a good solution for real industrial applications. During temperature measurement, care must be taken to ensure that the heating and cooling effects of weather parameters are shielded as little as possible due to the sensor housing.

The sensor’s power supply must be such that it can withstand the adversities of the environment and have a minimal impact on operational safety. A valuable solution can be hybrid solutions that combine the advantages of batteries and current transformers.

Finally, proper communication and EMC protection must be ensured, which can significantly increase the device’s reliability. 

By creating such a sensing device, all critical points of transmission lines can be monitored at low costs, which enables flexible and safe commissioning for TSOs.

## 6. Conclusions

The sensor monitoring of high-voltage transmission lines is increasingly becoming a concern of TSOs. A well-designed line-monitoring system can help increase transfer capacity via a DLR method, provide data for extensive asset management and health monitoring, and support those subsystems whose primary purpose is to reduce the damage caused by extreme weather phenomena (icing, forest fire, etc.). 

This paper focused on the thermal monitoring strategies of the phase conductors of high-voltage lines. At the beginning of the paper, the directions that determined sensor installation strategies in the past were presented. Although the detailed approaches theoretically contain many possible solutions for thermal monitoring, it is essential to emphasize that no universally accepted sensor placement strategy integrates all critical aspects simultaneously. 

In the second part of the paper, a new sensor placement concept that would compensate for the shortcomings of previous models was presented in three steps. Through the technical analysis of the transmission line, in Step 1, the tension section whose monitoring is essential is selected. Step 2 reveals the cause of the thermal constraint and presents a new type of tension-section-ranking method to provide an order of the sections to be equipped with devices if one sensor is insufficient on the line. Step 3 shows the chance of thermal overload in other tension sections due to the spatial change in weather parameters. One significant advantage of this concept is that it can assign a risk factor to the different number of sensor solutions. This provides extra information for system operators. 

The operation of this novel concept was presented in the case of a 220 kV double-circuit power line. The simulation results revealed that the number of sensors was influenced by the data-sampling frequency of the devices. It was also found that if the goal was to avoid annealing, more sensors were necessary to avoid clearance problems. Additionally, our simulations showed the tension sections whose monitoring would not be economically justified. On the other hand, the simulations showed that one or two sensors did not result in safe operation on the high-voltage grid. 

Although the distributed sensor installation protocol is essential in some cases, it significantly increases the costs of line-monitoring systems. One way to reduce costs is to develop sensors that can be produced at a low cost. These types of sensor devices not only significantly reduce the costs of line-monitoring systems, but with their help, TSOs have more and more reliable data available, which results in more flexible operation and safer energy transmission.

## Figures and Tables

**Table 1:** Safety factors as a function of sampling frequency and number of sensors [16].

Sensor No.	10 min Sampling		15 min Sampling		20 min Sampling	
	Clearance	Annealing	Clearance	Annealing	Clearance	Annealing
1	68.61%	78.48%	61.35%	71.06%	58.85%	68.14%
2	83.17%	92.61%	80.91%	87.30%	78.29%	84.76%
3	93.71%	96.60%	89.00%	93.03%	86.78%	91.07%
4	97.90%	98.58%	95.10%	96.32%	93.47%	94.89%
5	99.00%	99.23%	97.10%	97.60%	95.79%	96.44%
6	99.75%	99.53%	98.94%	98.37%	98.19%	97.43%
7	99.95%	99.78%	99.67%	99.10%	99.35%	98.52%
8	99.99%	99.90%	99.86%	99.40%	99.68%	98.94%
9	99.99%	99.96%	99.97%	99.55%	99.90%	99.16%
10	99.99%	99.99%	99.99%	99.78%	99.95%	99.44%
11	99.99%	99.99%	99.99%	99.90%	99.99%	99.58%

## Data Availability

The data that support the findings of this study are available from the corresponding author (L.R.) upon reasonable request.
